# Cross-sectional association between vitamin B12 status and probable postpartum depression in Indian women

**DOI:** 10.1186/s12884-021-03622-x

**Published:** 2021-02-17

**Authors:** Pooja Dhiman, Raji Ramachandran Pillai, Anand Babu Wilson, Nancy Premkumar, Balaji Bharadwaj, Veena P. Ranjan, Soundravally Rajendiran

**Affiliations:** 1 Department of Biochemistry, JIPMER, Puducherry, India; 2 Medico-Social Wing, JIPMER, Puducherry, India; 3 Department of Psychiatry, JIPMER, Puducherry, India; 4 Department of Obstetrics & Gynecology, JIPMER, Puducherry, India

**Keywords:** Vitamin B12, Folate, Postpartum depression

## Abstract

**Background:**

Vitamin B12 is an essential micronutrient for neurological function, as it leads to the regeneration of methionine from homocysteine, which is precursor of biologically active molecule S-Adenosyl Methionine (SAM). Pregnancy is a state of increased demand and delayed postpartum repletion of nutrients may predispose women to depression.

**Methods:**

We included women who visited the hospital at 6-weeks postpartum for a regular checkup. Inclusion criteria were age (18–50 years), and willingness to donate venous sample for analysis. Exclusion criteria included previous history of mood disorders or antidepressant medication use, and any systemic illness like hypothyroidism, epilepsy, diabetes, and hypertension. Based on EPDS score of 10 as a cutoff, 217 women with probable postpartum depression (PPD) and equal number of age and BMI matched controls were included.

Plasma total vitamin B12, holotranscobalamin (holotc), homocysteine (hcy), methyl malonic acid (MMA), 5-methyl tetrahydrofolate (THF), SAM and serotonin levels were estimated using commercially available ELISA kits. Combined B12 (cB12) score was calculated from study parameters. Multivariate analysis was performed to assess the risk of probable postpartum depression.

**Results:**

Total vitamin B12 and combined B12 score were found to be significantly lower (*p* = 0.001) and MMA (*p* = 0.002) and 5-methyl THF (*p* < 0.001) levels were higher in women with probable depression than women without probable PPD. Women in the lowest vitamin B12 quartile had 4.53 times higher likelihood of probable postpartum depression (*p* < 0.001). Multivariate analysis demonstrated that decreasing vitamin B12 (OR = 0.394; 95% CI: 0.189–0.822) and cB12 (OR = 0.293; 95% CI: 0182–0.470) and increasing MMA (OR = 2.14; 95% CI: 1.63–2.83) and 5-methyl THF levels (OR = 3.29; 95% CI: 1.59–6.83) were significantly associated with the risk of probable PPD.

**Conclusion:**

Low vitamin B12 may contribute to depressive symptoms in vulnerable postpartum period.

## Background

Nutritional deficiencies have been linked with depressive symptoms [1]. Numerous studies have explored the relationship of nutritional factors like omega-3, fatty acids, zinc, B-vitamins with perinatal depression [2]. Lower levels of vitamin B12 have been suggested to mediate depression in general population [3] and during the perinatal period [2].

Vitamin B12 is involved in methionine regeneration and methylation reactions, which occur at a high rate during fetal development, and influence the synthesis and neuronal availability of serotonin/ 5- hydroxyl tryptamine (5-HT). If B12 is deficient, it renders a low bioavailability of 5-HT, and may lead to depression. Apart from its direct effect, an increase in homocysteine (hcy), which accumulates in vitamin B12 deficiency can precipitate depressed mood, by increasing reactive oxygen species and further inducing neuronal apoptosis [4]. Deficiencies in vitamin B12 can become overt during pregnancy and lactation, when demands of growing fetus and delayed repletion impose significant nutritional demand [5, 6].

Postpartum depression (PPD) is defined as an episode of depression within 6 weeks of delivery [7], which is reported to have a global prevalence of 10–15 and 19–22% in India [8]. PPD has far reaching physical, mental and social implications in women, extending to her child and the nearest family. Impaired interaction and delayed physical and mental growth of the child has been observed in depressed mothers.

Effect of vitamin B12 deficiency on mood can be explained its direct and indirect cellular effects. As an indirect effect of vitamin B12 deficiency, there is accumulation of 5-methyl THF, which additionally hampers the nucleotide synthesis and leads to genomic instability [9]. Additionally, vitamin B12 deficiency may hamper conversion of MMA to succinyl CoA as well as affect myelination [10]. Recent reports also suggest vitamin B12 deficiency to be associated with impaired glutathione peroxidase activity and increase in free radicals [11]. To date, few studies have explored the association of vitamin B12 and its metabolites with PPD. there are reports of association of vitamin B12 with PPD where dietary intake was measured but not plasma levels. Miyake et al. observed no measurable association between intake of vitamin B12 and the risk of postpartum depression in 865 Japanese women [12]. Blunden et al. (2012) in Britain collected the information on vitamin B12 intake and EPDS in 2856 women in pregnancy and followed them up to 1 year postpartum. They concluded that there is no significant association of vitamin B12 intake and risk of postpartum depression [13].

As the reference intake of vitamin B12 increases from 2.4mcg in non-pregnant state to 2.6mcg in pregnancy and 2.8mcg in lactation, its nutritional reserves are expected to decline in postpartum period especially when the women already has lower reserves of vitamin B12 as observed in India [14].

Taking these facts together, we hypothesize that low vitamin B12 levels are associated with postpartum depression in our population. Therefore, this study aims to explore the association between vitamin B12 and probable PPD in South Indian population.

## Methods

### Study design

**Fig. 1:**
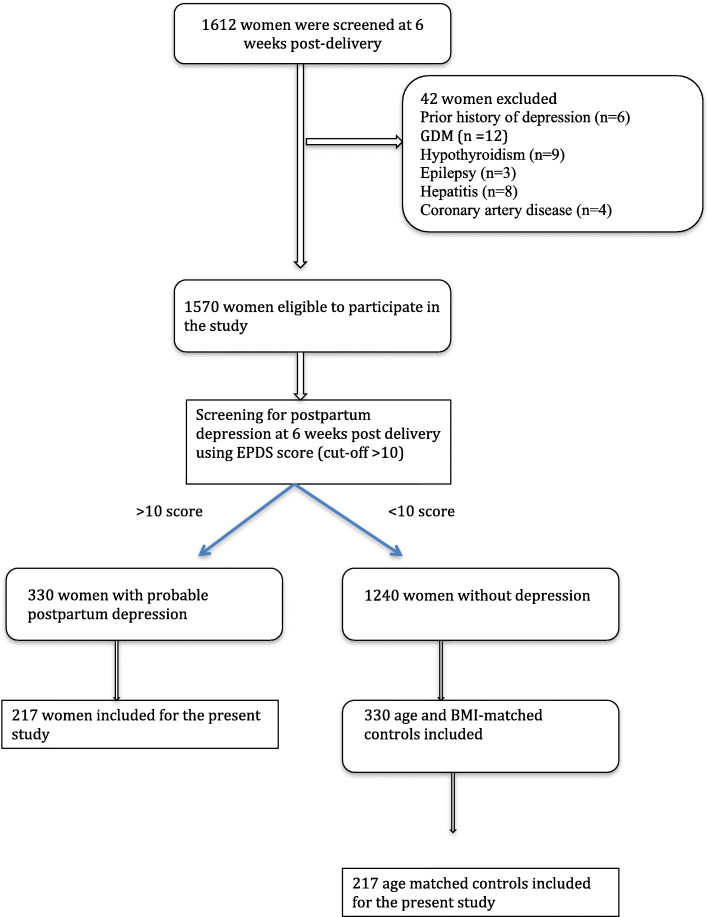
Summary of process for the selection and recruitment of study subjects

This cross-sectional study was conducted using archived plasma samples from a previous study [15]. The study was approved by Institute Ethics Committee (JIP/IEC/2014/5/319) and was conducted in accordance with the Declaration of Helsinki. Written informed consent was obtained from all the women included in the study. Briefly, women who delivered in Jawaharlal Institute of Postgraduate Medical Education & Research (JIPMER), Puducherry, India, a tertiary care hospital in the south of India, and visited the hospital at 6-weeks postpartum for a regular checkup, were recruited from January 2014 to December 2017. Inclusion criteria included age between 18 and 50 years, and willingness to donate venous sample for analysis. Women with previous history of mood disorders or antidepressant medication usage were excluded from the study. The parent study was conducted in a sample size of 660 women. The detailed procedure of exclusion and inclusion of women is described in the flow chart (Fig. 1). The socio-demographic data and nutritional profile of the women was recorded using semi-structured questionnaire, which was internally validated. Details of the anthropometric, obstetric and routine laboratory characteristics of the women, along with neonatal birth weight, APGAR score at 1 and 5 min and crying behavior by Wessel’s index [16] were noted from their medical records.

Edinburg Postnatal Depression Scale (EPDS), both in English and transliterated version in Tamil, was used for the screening of depression by trained medical social worker at 6-weeks postpartum. This excluded women experiencing postpartum blues – a commonly experienced self-limiting mild depressive symptoms seen between 2 and 5 days post delivery [17]. Study by Benjamin et al. for our population demonstrated a cut-off of 8/9 to have a sensitivity of 94.1% and specificity of 90.1% for detecting PPD in South Indian population in a setting of Primary health care [18]. Since this study was conducted in a tertiary care center, a cut-off of ≥10 was used to identify women as probable PPD as suggested by experts from psychiatry department.

At 6 weeks postpartum, 5 ml of whole blood was collected in EDTA vial. For biochemical parameters, 3 ml of sample was further centrifuged at 3000 rpm at room temperature for 10 min to obtain plasma, which was stored at − 80 °C until used.

For the present study, samples were included based on reconsent obtained from the women, and the quality and quantity of archived samples, yielding a final sample size of 434. Based on Edinburgh Postpartum Depression Scale (EPDS) consistent with probable PPD (EPDS > 10; *n* = 217 women) and an equal number of age-matched controls (*n* = 217) were taken.

The power calculation was carried out using online software (Open Source Epidemiologic Statistics for Public Health, Version 3.01, Emory University, and Rollins School of Public Health). We calculated the sample size using mean and standard deviation of plasma vitamin B12 levels in postpartum depression reported earlier by Chong et al [19] The estimated sample size was 217 subjects in each group (total 434 subjects), at 5% level of significance and 80% power.

### Maternal vitamin B12 metabolites in circulation

Plasma levels of total vitamin B12 (Bioassay Technology Lab, China), holotranscobalamin (holotc; Fine test, Wuhan, China), Methylmalonic acid (MMA; Bioassay Technology Lab, China), homocysteine (hcy; Bioassay Technology Lab, China), 5-methyl THF (Cloud-Clone Corp, USA), S- adenosyl methionine (SAM; Bioassay Technology Lab, China) and serotonin (Bioassay Technology Lab, China) were measured using commercially available ELISA kits. The minimum detectable limits are: total vitamin B12 (2.38 pmol/L), holotc (0.94 ng/ml), MMA (0.02 ng/ml), hcy (0.092 nmol/L), 5-methyl THF (0.43 ng/ml), SAM (2.63 ng/ml), and serotonin (1.22 ng/ml), with overall intra-assay coefficient of variation (CV) of< 8% and inter-assay CV of < 10%. We also calculated a combined indicator of cobalamine deficiency (cB12), described by Fedosov et al., which considers four parameters: total vitamin B12, holotc, hcy and MMA along with age of the women [20].

### Data analysis

All statistical analysis was performed using SPSS version 20.0 (SPSS Inc., Chicago, Il, USA). Normally distributed data was described as mean (SD), non-normal data as median (IQR) and categorical data was described as percentages. Mann-Whitney U test was used to find difference between the two groups in case of continuous variables, as they were in non-normal distribution and chi-square was used for categorical variable.

Linear regression analyses were performed using EPDS score as dependent variable and log transformed total vitamin B12, holotc, 5-methyl THF, hcy, SAM, MMA, and serotonin as independent variables. Linear regression for cB12 was performed independently from other parameters. Multiple logistic regression analysis with different EPDS score cut-off groups were performed using log-transformed values of circulating markers. Linear regression and multiple logistic regression outcomes were observed in both unadjusted and adjusted mode (for both nutritional and socio-demographic characteristics which were found to be significantly differing between two groups). To further examine the dose-response, plasma vitamin B12, 5-methyl THF and MMA were modeled as quartiles and logistic regression analysis was used as above. The measures of association, odds ratio (OR), with its corresponding 95% confidence intervals was reported and a two-tailed *p*-value of less than 0.05 were considered statistically significant. Path analysis was carried out using SPSS AMOS (version 26) (trial version) to determine if the association of total vitamin B12 to postpartum depressive symptoms was mediated through elevated MMA or 5-methyl THF levels.

## Results

### Population characteristics

Mothers with probable postpartum depression were more likely to belong to middle socioeconomic group (*p* = 0.002), have had more than one child (*p* = 0.002), were dissatisfied with their marriage (*p* < 0.001) & the gender of the child (*p* = 0.004), have had an unplanned pregnancy (*p* < 0.001), and have had higher rates of cesarean section (*p* = 0.014). They also reported lower intake of milk (*p* < 0.001), meat (*p* = 0.012) and eggs (*p* = 0.002) during the postpartum period. Age, BMI, other maternity stressor (e.g., lack of sleep) did not differ between those with and without postpartum depression (Table 1). All the women included in the study reported intake of folate supplementation during pregnancy, which was supplied by the hospital as a routine care.

**Table 1:** Maternal anthropometric, social, obstetrical and psychological characteristics in 434 women with and without probable PPD according to EPDS score cut-off of > 10 (N, %, mean and SD)

Variable	Group		***P***-value
	***Women with probable postpartum*** ***depression (n = 217)***	***Women without probable postpartum depression (n = 217)***	
**Anthropometric characteristics**			
Age (years)			
Mean (SD)	26.22 (4.30)	25.56 (4.08)	0.102^
Min – Max	18–40	19–36	
Age			
< =25 yrs	111 (51.2)	118 (54.4)	0.785^@^
26–30 yrs	77 (35.5)	73 (33.6)	
> 30 yrs	29 (13.4)	26 (12.0)	
BMI (kg/m^2^)			
Mean (SD)	25.08 (4.55)	25.61 (4.57)	0.225^
Min – Max	13.33–39.12	13.90–40.50	
BMI (kg/m^2^)			
< =18.5	12 (5.5)	6 (2.8)	0.523^@^
> 18.5–24.9	109 (50.2)	109 (50.2)	
> 24.9–29.9	60 (27.7)	62 (28.6)	
> 30	36 (16.6)	40 (18.4)	
**Socio-demographic characteristics**			
Family structure			
Extended	149 (68.7)	150 (69.1)	0.917^@^
Nuclear	68 (31.3)	67 (30.8)	
SES^a^			
Lower	4 (1.8)	9 (4.2)	0.002^@^
Upper lower	112 (51.6)	142 (65.4)	
Lower middle	54 (24.9)	31 (14.3)	
Upper middle	47 (21.7)	32 (14.8)	
Upper	–	3 (1.4)	
Total children			
1	116 (53.5)	148 (68.2)	0.002^@^
> =2	101 (46.5)	69 (31.8)	
Marital dissatisfaction			
No	153 (70.5)	213 (98.2)	< 0.001^@^
Yes	64 (29.5)	4 (1.8)	
Any death in the family in the last one year			
No	213 (98.2)	215 (99.1)	0.411^@^
Yes	4 (1.8)	2 (0.9)	
Breast feeding			
Yes	214 (98.6)	215 (99.1)	0.653^@^
No	3 (1.4)	2 (0.9)	
Sleeping hours after delivery			
< 6 h/day	100 (46.1)	98 (45.2)	0.847^@^
> 6 h/day	117 (53.9)	119 (54.8)	
Are you happy with the baby gender			
Yes	46 (21.2)	24 (11.1)	0.004^@^
No	171 (78.8)	193 (88.9)	
Family members happy with the sex of the child			
No	55 (25.4)	49 (22.6)	0.500^@^
Yes	162 (74.6)	168 (77.4)	
**Obstetrics characteristics**			
Parity			
Primi	102 (47.0)	120 (55.3)	0.084^@^
Multi	115 (53.0)	97 (44.7)	
History of miscarriage/still birth			
No	165 (76.0)	165 (76.0)	0.087^@^
Miscarriage	51 (23.5)	45 (20.7)	
Still birth	1 (0.5)	7 (3.2)	
Pregnancy planning			
Unplanned	108 (49.8)	66 (30.4)	< 0.001^@^
Planned	109 (50.2)	151 (69.6)	
Delivery type			
Vaginal	135 (62.2)	163 (75.1)	0.014^@^
Vaginal assisted	17 (7.8)	10 (4.6)	
Caesarean	65 (30.0)	44 (20.3)	
Difficulty in delivery			
Yes	86 (39.6)	77 (35.5)	0.372^@^
No	131 (60.4)	140 (64.5)	
Perinatal complications			
Yes	18 (8.3)	18 (8.3)	0.999^@^
No	199 (91.7)	199 (91.7)	
**Nutritional characteristics**			
Diet			
Veg	11 (5.1)	13 (6.0)	0.674^@^
Mixed diet	206 (94.9)	204 (94.0)	
Milk			
Seldom	7 (3.2)	9 (4.2)	< 0.001^@^
Once per day	86 (39.8)	41 (18.9)	
2–3 times per day	109 (50.5)	148 (68.2)	
> 4 times per day	14 (6.5)	19 (8.8)	
Curd/Butter milk			
Seldom	178 (82.0)	196 (90.3)	0.002^@^
1–2 times per week	11 (5.1)	13 (6.0)	
3–4 times per week	11 (5.1)	6 (2.8)	
Once a day	17 (7.8)	2 (0.9)	
Fishes			
Seldom	65 (30.4)	64 (29.6)	0.808^@^
1–2 times per week	83 (38.8)	87 (40.3)	
3–4 times per week	54 (25.2)	57 (26.4)	
Once a day	12 (5.6)	8 (3.7)	
Meat			
Seldom	85 (39.7)	74 (34.3)	0.012
1–2 times per week	88 (41.1)	88 (40.7)	
3–4 times per week	25 (11.7)	47 (21.8)	
Once a day	16 (7.5)	7 (3.2)	
Egg			
Seldom	38 (17.7)	32 (14.8)	< 0.001^@^
1–2 times per week	36 (16.7)	100 (46.3)	
3–4 times per week	55 (25.6)	68 (31.5)	
Once a day	86 (40.0)	16 (7.4)	
Term baby			
Pre-term	34 (15.7)	24 (11.1)	0.158^@^
Term	183 (84.3)	193 (88.9)	
Gender of baby			
Male	121 (55.8)	133 (61.3)	0.242^@^
Female	96 (44.2)	84 (38.7)	

^a^Based on Kuppusamy’s Socioeconomic scale [21]

^@^ *P*-value based on Chi-square test

^ *P*-value based on Mann-Whitney U test

### Biochemical analysis

The median total vitamin B12 levels in cases were significantly lower than controls with no difference in its active form, holotc. MMA, a marker of functional deficiency of vitamin B12 was significantly elevated in depressed women compared those without depression (*p* = 0.002) (Table 2). Though hcy, a non-specific marker of vitamin B12 deficiency was notably increased in cases than control, the difference did not achieve a statistical significance (*p* = 0.057). Also, 5-methyl THF was increased in depressed women (*p* < 0.0001) signifying a ‘folate trap’ due to existing B12 deficiency. A significantly lower value of cB12 was observed in women with probable depression as compared to controls (*p* < 0.001).

**Table 2:** Plasma levels of vitamin B12, and other metabolites in study population

Characteristics	Control median (IQR)	Cases median (IQR)	***P*** value
Total vitamin B12 (pg/ml)	353.9 (284.4–415.9)	294 (247.7–365.8)	< 0.001
Holotranscobalamin (pmol/L)	57.4 (49.4–86.7)	60.9 (52.3–74.4)	0.510
Homocysteine (micromol/L)	3.02 (1.9–4.52)	2.99 (2.39–5.52)	0.057
MMA (micromol/L)	19.1 (16.61–23.05)	22.09 (14.27–124.49)	0.002
cB12	1.59 (−0.94–2.69)	1.25 (−0.79–2.7)	< 0.001
SAM (ng/ml)	32.89 (20.01–73.02)	38.47 (22.54–64)	0.373
5 methyl THF (ng/ml)	2.52 (2.08–3.25)	2.93 (2.39–3.67)	< 0.001

Women in the study were divided into 3 groups based on clinical decision limits of total vitamin B12: Deficient (less than 150 pmol/L), low normal (190–300 pmol/L) and Sufficient (> 300 pmol/L). In the whole cohort (*n* = 434), 58% of the women had sufficient vitamin B12, 38.5% had low normal levels and 3.5% women were overtly deficient. Among cases and controls, significantly higher number of women with PPD had low normal vitamin B12 (49.8%) compared to women without PPD (27.2%). The cutoff limit for holotranscobalamin is 35 pmol/L, to define sufficient and deficient status. There was no significant difference among women with and without depression for clinical decision limits of holotranscobalamin.

### Association of vitamin B12 and its deficiency indicators with probable postpartum depression

Regression analyses showed negative association of vitamin B12, cB12 and serotonin with probable PPD, whereas a positive association of MMA, hcy and 5-methyl THF was observed with depressive symptoms (Table 3).

**Table 3:** Association of Edinburg Postpartum Depression Scale (EPDS) scores with maternal circulating parameters (log transformed)

Model	Vitamin B12 metabolites	***Β***	***p***-value
Unadjusted	Total B12	−0.103	< 0.027
	MMA	0.263	< 0.001
	5-methyl THF	0.157	0.001
	Hcy	0.140	0.015
	holotc	0.979	0.922
	SAM	−0.098	0.085
	Serotonin	−0.124	< 0.040
	cB12	−0.276	< 0.0001
Adjusted^a^	Total B12	−0.064	0.146
	MMA	0.161	0.001
	5-methyl THF	0.118	0.010
	Hcy	0.155	0.005
	holotc	−0.051	0.260
	SAM	− 0.067	0.218
	Serotonin	−0.161	0.005
	cB12	0.169	< 0.001

^a^Adjusted for socioeconomic status, marital dissatisfaction, unplanned pregnancy and type of delivery

Regression model after adjusting for significant socio-demographic and nutritional characteristics demonstrated that the likelihood of probable postpartum depression decrease by a factor of 0.39 for every unit increase in total vitamin B12 (OR = 0.394; 95% CI: 0.189–0.822) and by a factor of 0.29(OR = 0.293; 95% CI:0182–0.470) for cB12. Both MMA and 5-methyl THF were found be significant predictors of probable postpartum depression (Table 4). SAM and hcy although were associated with probable PPD in unadjusted model, on subsequent adjustment with nutritional and socio-demographic covariates, lost that significance (Table 4).

**Table 4:** Logistic regression model of the association of maternal circulating vitamin B12 deficiency markers with probable postpartum depression based on Edinburg Postpartum Depression Scale (EPDS) cut-off of > 10

Model	Vitamin B12 metabolites	Odds ratio	95% CI	***p***-value	Predictive value of the model
Unadjusted	Total B12	0.355	0.175–0.718	0.004	76.5%
	MMA	1.99	1.53–2.59	< 0.001	
	5-methyl THF	2.96	1.46–6.01	0.003	
	Hcy	1.45	1.00–2.09	0.048	
	holotc	0.979	0.641–1.497	0.922	
	SAM	0.74	0.573–0.967	0.027	
	Serotonin	0.844	0.581–1.22	0.372	
	cB12	0.357	0.239–0.532	< 0.0001	
Adjusted^a^	Total B12	0.514	0.289–0.912	0.009	78.6%
	MMA	2.04	1.53–2.11	< 0.001	
	5-methyl THF	3.18	1.42–6.08	0.001	
	Hcy	1.42	0.959–2.1	0.079	
	holotc	0.968	0.61–1.49	0.893	
	SAM	0.762	0.59–1.01	0.063	
	Serotonin	0.832	0.573–1.21	0.309	
	cB12	0.312	0.192–0.501	< 0.001	

^a^Adjusted for socioeconomic status, marital dissatisfaction, unplanned pregnancy and type of delivery

In quartile analysis, decreasing total vitamin B12 levels were associated with increasing odds of probable postpartum depression. With the highest vitamin B12 quartile as reference, those in the lowest vitamin B12 quartile had 4.53 times higher likelihood of probable postpartum depression (*p* < 0.001). Increasing MMA and 5-methyl THF levels were also significantly associated with the risk of PPD (OR = 3.26 and 2.97, respectively). No associations were observed between quartiles levels of hcy, SAM and probable postpartum depression.

On path analysis, the model with total vitamin B12 as the predictor, MMA the mediator and EPDS score as the dependent variable was statistically significant (*p* < 0.001). This indicates the role of MMA as a potential mediator in appearance of depressive symptoms in postpartum period. On the other hand, 5-methyl THF as a mediator for the effect of vitamin B12 deficiency on EPDS score was not found to be significant. Mediation analysis with serotonin and SAM was not statistically significant.

## Discussion

As hypothesized in this study, women with probable PPD were observed to have low vitamin B12 levels and higher MMA and 5-methyl-THF levels compared to non-PPD women. Holotranscobalamin, homocysteine and SAM levels were observed not to be different between groups.

Our findings are partially consistent with few studies, which focused on the role of nutritional deficiencies, especially vitamin B12, in the development of postpartum depression. Study by Abou-Saleh et al. observed lower vitamin B12 concentration in women with depressive symptoms (*n* = 62) at 7th day postpartum, and reported a significant correlation between EPDS score and vitamin B12 levels (*r* = 0.39; *p* < 0.01) [22]. In contrast to our data, Chong et al. found no association between vitamin B12 levels and postpartum depression [19]. However the difference in the study period i.e. 26th–28th week of gestation in Chong et al. versus 6th week postpartum in the present study might be a factor for this difference in the observation. We also observed that compared to controls more women with PPD had low normal vitamin B12 (49.8% versus 27.2% in controls; *P* = 0.001). Similar findings in ante-natal depression is reported recently in 174 pregnant women using secondary data analysis of National Health and Nutrition Examination Survey data, CDC, in USA [23]. They observed higher frequency of antenatal depression in women with low normal vitamin B12 (*n* = 9; 15.2%) compared with those with sufficient vitamin B12 (*n* = 6; 5.2%). Lukose et al. from Bangalore, India also explored the status of vitamin B12 with antepartum depression in 365 pregnant women in their 1st trimester [24]. Although they observed 51% of women to be vitamin B12 deficient (< 150 pmol/L), they observed no significant association of vitamin B12 with antenatal depression. Differences in depression screening tool (Kessler Psychological Distress Scale versus EPDS) and time of sample collection (antepartum versus postpartum) may explain the divergent results observed in the two studies. Other significant studies exploring the association between B-vitamin and PPD measured only dietary intake of folate, vitamin B6 and B12 [12] or supplementation [25–27] without measuring the circulating levels of vitamin B12 [13, 28, 29]. We conducted a pilot study to observe the status of vitamin B12, total folate and hcy in 24–48 h post delivery, and found higher hcy levels in depressed women when compared to the non-depressed. Of these 108 women, 28 came back at 6 weeks postpartum where a similar result was observed. As the drop out rate in previous study was 80%, it may not truly reflect the vitamin B12 status at 6 weeks postpartum [30]. As many of these studies are observational, it is not possible to draw any causal inference.

The association between low vitamin B12 levels with depressive symptoms has also been observed in general population. Tiemeier et al. observed low vitamin B12 and high homocysteine to be associated with depression in elderly women [31]. Similarly, Penninx et al. observed vitamin B12 deficiency to be associated with two-fold increased risk for severe depression in community dwelling physically disabled women [32]. A community based observational study in elderly population by Robinson et al. found lower levels of both total vitamin B12 and holotranscobalamin to be associated with depressive symptoms [33].

We observed no significant difference in holotc levels between the two groups despite lower total vitamin B12 levels in women with PPD. This finding is in contrast to previous reports where in women with low vitamin B12 (150-300 pmol/L), holotc was found to be decreased in spite of normal total vitamin B12 levels [34]. The failure of holotc to show any significant change despite decreased total vitamin B12 levels indicates the dynamicity of this molecule to such an extent that it became a poor marker of B12 deficiency and similar finding has been reported previously [35].

Combined measurement of Vitamin B12, as reported by Sergey et al., provides a comprehensive assessment of vitamin B12 status. We found significant difference between cB12 in two groups despite the fact that holotc and hcy were not different between them. These results emphasize the role of combined markers to measure B12 deficiency especially in postpartum period where the metabolic status is dynamic. Campos et al. in their recent study in 11,833 samples observed individual markers of vitamin B12 deficiency (total vitamin B12, hcy, holotc, MMA) to be in agreement with cB12 scores [36]. Combined B12 score has also been observed to be associated with symptoms of B12 deficiency like a decrease in hemoglobin concentration, cognitive functions and peripheral nerve conductivity [37]. The utility of cB12 in detecting vitamin B12 deficiency in pregnancy and postpartum period needs to be further evaluated.

We observed a positive association of MMA with EPDS scores, which signifies the functional deficiency of vitamin B12 in PPD. Similar results, have been reported in general depression in elderly age group [31–33]. Path analysis demonstrated that the effects of low vitamin B12 on depressive symptoms were direct and mediated by MMA, but not 5-methyl THF. These results reflect a vital role of MMA in mediating the cellular effects of vitamin B12 deficiency. High MMA levels can competitively inhibit succinate dehydrogenase enzyme, which further inhibits mitochondrial oxidation and may lead to neuronal damage [38]. Also, MMA hampers the activity of glycine cleavage system, leading to the accumulation of glycine, an inhibitory neurotransmitter. Recently, Li et al. reported that high MMA levels cause neuronal apoptosis via micro-RNA miR-9 [39], which adds another dimension to the cellular effect of high MMA levels.

A noteworthy elevation of 5-methyl THF levels in the group with depressive symptoms probably indicates cellular folate trap. Our findings are in concordance with Selhub et al. study, who reported that low vitamin B12 levels with higher blood folate interferes with both regeneration of homocysteine to methionine and isomerization of methyl malonyl CoA (MMA) to succcinyl CoA, leading to increased circulating levels of both homocysteine and MMA [40]. Also, the possibility of high folate concentration either interfering with the distribution of vitamin B12 between cytosol and mitochondria or with the catalytic activity of methionine synthase and methyl malonyl CoA mutase enzymes has been proposed.

A strength of this study is the usage of a representative sample of women presenting at a tertiary care hospital coupled with the measurement of vitamin B12, its active metabolites, and active levels of folate (i.e., 5-methyl THF) at a 6-week postpartum period in a folate-supplemented population. Another strength is the consideration of nutritional and socio-demographic covariates, enabling observations to be quantified independent of folate. A limitation of this study is the retrospective nature of the data collection and the secondary nature of the analysis. Additionally, the use of a self-reported screening measure of PPD (i.e., EPDS) poses potential limitation on the lack of a clinical diagnosis of PPD.

This study concludes that low vitamin B12 & cB12 and high MMA & 5-methyl THF are predictors of probable PPD. Since MMA is a sensitive marker of cellular B12 deficiency and has been shown to mediate the effects of low vitamin B12 on depressive symptoms in our study, it may be used as a tool for judging the status of vitamin B12 in postpartum period. Further research is necessary to understand the biochemical link between MMA and 5-methyl THF with depression. Our findings support vitamin B12 supplementation during pregnancy, along with routine folic acid, as a prophylactic measure against postpartum depressive symptoms. A recommendation consistent with a systematic review that demonstrated women who consume multivitamin supplementation during pregnancy may avoid depressive symptoms in perinatal period [41]. To substantiate these suggestions, it is necessary to perform multi-centric randomized controlled trials in India.

## Data Availability

The data and material are available with the corresponding author on request.

## Abbreviations

PPD: Postpartum depression
EPDS: Edinburg Postpartum depression Scale
Hcy: Homocysteine
Holotc: Holotranscobalamine
MMA: Methylmalonic acid
SAM: S-adenosyl methionine
